# Malignant mammary tumor in female dogs: environmental contaminants

**DOI:** 10.1186/1746-1596-5-45

**Published:** 2010-06-30

**Authors:** Fábio HE Andrade, Fernanda C Figueiroa, Paulo RO Bersano, Denise Z Bissacot, Noeme S Rocha

**Affiliations:** 1 Maranhão State University (UEMA), São Luis, MA, Brazil; 2 School of Veterinary Medicine and Animal Science, São Paulo State University (UNESP) - Botucatu, SP, Brazil; 3 CEATOX - Botucatu Bioscience Institute (UNESP) - Botucatu, SP, Brazil

## Abstract

Mammary tumors of female dogs have greatly increased in recent years, thus demanding rapid diagnosis and effective treatment in order to determine the animal survival. There is considerable scientific interest in the possible role of environmental contaminants in the etiology of mammary tumors, specifically in relation to synthetic chemical substances released into the environment to which living beings are either directly or indirectly exposed. In this study, the presence of pyrethroid insecticide was observed in adjacent adipose tissue of canine mammary tumor. High Precision Liquid Chromatography - HPLC was adapted to detect and identify environmental contaminants in adipose tissue adjacent to malignant mammary tumor in nine female dogs, without predilection for breed or age. After surgery, masses were carefully examined for malignant neoplastic lesions. Five grams of adipose tissue adjacent to the tumor were collected to detect of environmental contaminants. The identified pyrethroids were allethrin, cyhalothrin, cypermethrin, deltamethrin and tetramethrin, with a contamination level of 33.3%. Histopathology demonstrated six female dogs (66.7%) as having complex carcinoma and three (33.3%) with simple carcinoma. From these tumors, seven (77.8%) presented aggressiveness degree III and two (22.2%) degree I. Five tumors were positive for estrogen receptors in immunohistochemical analysis. The contamination level was observed in more aggressive tumors. This was the first report in which the level of environmental contaminants could be detected in adipose tissue of female dogs with malignant mammary tumor, by HPLC. Results suggest the possible involvement of pyrethroid in the canine mammary tumor carcinogenesis. Hence, the dog may be used as a sentinel animal for human breast cancer, since human beings share the same environment and basically have the same eating habits.

## Introduction

Currently, mammary tumors represent 50% of all neoplasms that afflict female dogs [1,2], and from these tumors 41 to 53% are of malignant character [3-5]. Epidemiological and clinical characteristics as well as biological behavior of such tumors in female dogs are similar to breast carcinomas in women, for this reason female dogs present an excellent comparative model to understand various aspects of carcinogenesis in both species [6]. It is believed that mammary neoplasias may occur as a result of complex interactions of distinct factors; however the exact cause is still under research. Through this dynamic process, the mammary tumor can be influenced by internal host factors such as genetics and external factors, including environmental contamination, that can enable or reduce the individual response [7-9]. The pyrethroids are among environmental contaminants the ones whose use has exponentially grown in recent years [10]. They are used to control pests in agriculture, ranching and domestic animals. The overspread use of this contaminant is associated with its efficiency in pest control and its relatively short half-life [11-13]. In humans and other animals, pyrethroids are readily absorbed cutaneously and also in the digestive and respiratory tracts. Once absorbed they are distributed to various tissues, but they are concentrated especially in the adipose tissue. International Agency for Research Cancer (IARC) includes agrotoxins, especially deltamethrin and cypermethrin in group 3 of risk level, that is, non-conclusive carcinogenic for humans, while the World Health Organization (WHO) classifies deltamethrin as a moderately dangerous insecticide [14,15]. Considering that this research line is still not used in regular basis for the veterinary medicine, the present study aimed to detect and identify levels of pyrethroid insecticides in adipose tissue adjacent to malignant mammary tumor in female dogs by the HPLC method and correlate these contaminants with the aggressiveness degree of the neoplasias.

## Material and Methods

**Figure 1:**
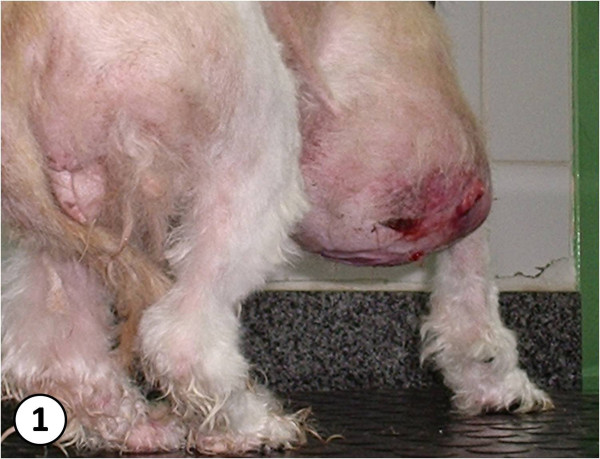
**great increase in right cranial mammary gland**.

**Figure 2:**
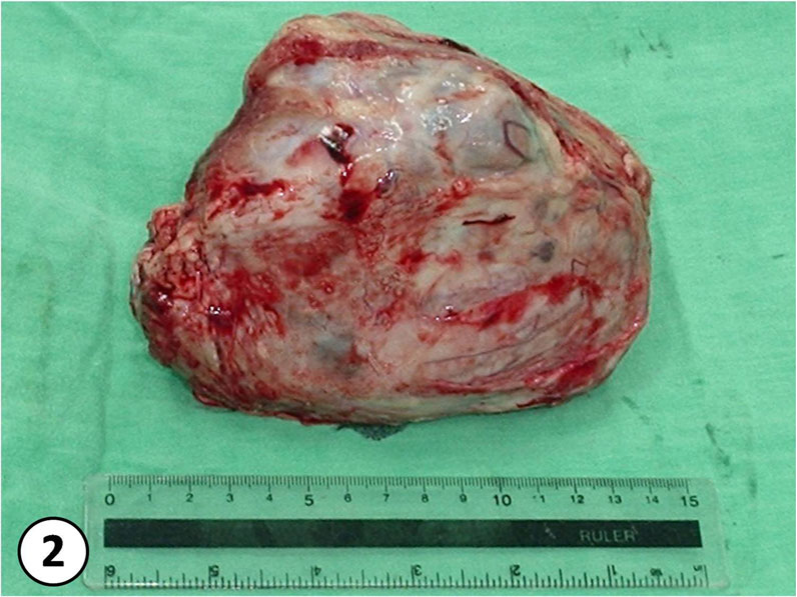
**tumor after unilateral mastectomy**.

Nine female dogs with mammary gland swelling (Fig. 1) were attended at the UNESP Veterinary Hospital in Botucatu, São Paulo - Brazil, and underwent mastectomy to excise the tumor (Fig. 2). Five grams of adipose tissue adjacent to the mammary tumor were analyzed by High Precision Liquid Chromatography - HPLC, following the Bissacot and Vassilieff method [16] (1997). Fragments of mammary tumor were fixed in 10% buffered formalin for 24 h, and then they were dehydrated in alcohol, diaphanized in xylene and put into paraffin. They were then cut into 3 μm-width fragments and stained with Hematoxylin-Eosin (HE). For the analysis of tumors it was used the Veterinary [17] and Human [18] classification. Immunohistochemical analysis followed the protocols from the Immunohistochemistry Laboratory from the Department of Clinical Veterinary Medicine of the School of Veterinary Medicine and Animal Science - UNESP - Botucatu. Antigen retrieval was carried out by microwave treatment in a 10 mM citrate buffer, pH 6.0. Tissue sections were incubated with primary monoclonal antibodies against ER (Novocastra - UK), clone LH2, in 1:40 dilution, incubated for 120 min and developed with polymer Novolink (Novocastra, UK). Tumors were considered positive when they presented more than 10% of nuclear marks from marked neoplasic cells. Graduations were set according to the intensity of positive marking as follows: (+) low intensity (++) mild intensity and (+++) high intensity described in previous studies [19].

## Results

**Figure 3:**
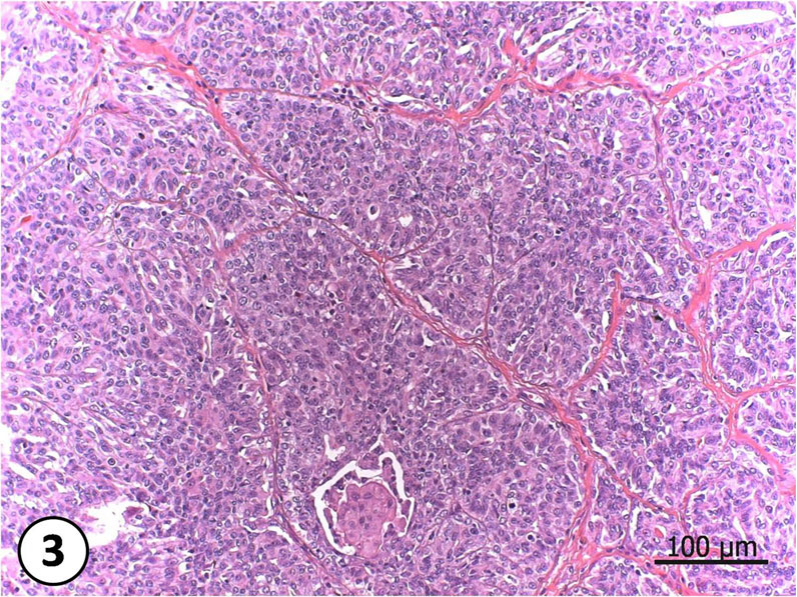
**Simple Carcinoma**. Neoplasia composed by proliferated epithelium cells, generating a hard standard with the loss of the glandular architecture.

**Figure 4:**
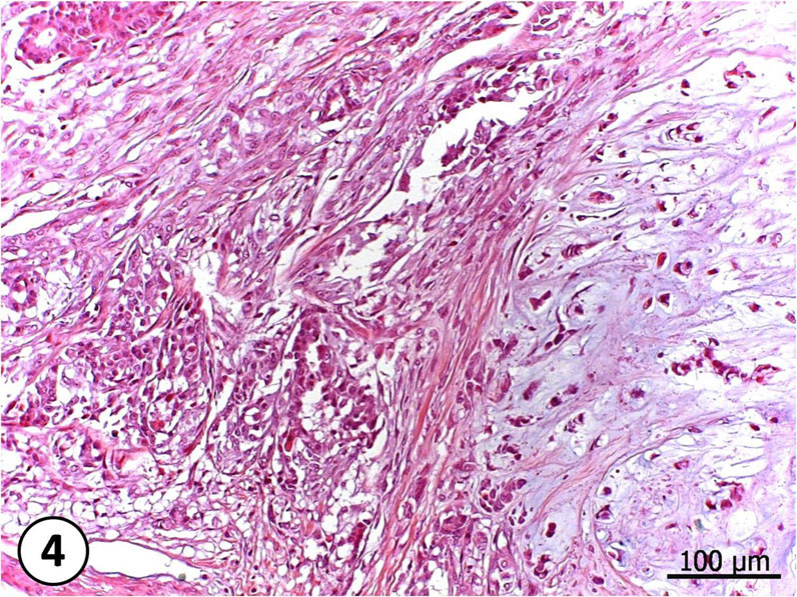
**Complex carcinoma**. Neoplasia composed by more than one cell type. Proliferated epithelial cells, with loss of gland architecture and presenting pleomorphism. Elongated mioeptithelial cells, presenting cartilagenous metaplasia and condroid matrix production. HE, 200X.

According to the Veterinary classification, from the nine examined tumors, three (33.3%) were classified as simple carcinoma (Fig. 3), and six (66.7%) as complex carcinoma (Fig. 4). But when it comes to the Human Medicine classification these nine cases were divided into: three (33.3%) simple carcinoma, being one tubulo-papilliferous, one ductal, one ductal infiltrative, and six (66.7%) metaplasic carcinomas. As for the histological malignity two tumors were degree I (22,2%) and seven tumors were degree III (77,8%). Receptors marking were positive for 5 animals, regardless of their mark intensity. From 9 analyzed female dogs, pyrethroids were detected in three animals distributed in the following manner: one (11.1%) female dog with 0.55 mg/g of deltamethrin and 0.32 mg/g of cyhalothrin; one (11.1%) female dog with 0.02 mg/g of deltamethrin and 0.05 mg/g of allethrin and one (11.1%) female dog with 0.03 mg/g of cypermethrin. Results are shown in table 1 and in the HPLC graphics (see Additional file 1).

**Table 1:** Veterinary and Human histopathological classification of Malignant mammary tumor in female dogs showing their malignity, positive immunemarking for estrogen receptors, and pyrethroid identification with their respective concentration levels in the analyzed samples

Classification - Carcinoma		Degree	Estrogen Receptor	HPLC	
			
Veterinary	Human			μg/g	Pyrethroid
Simple	Tubulo-papilliferous	III	+++	0.55 0.32	Deltamethrin Cyhalothrin
Complex	Metaplasic	I	0		--------------
Complex	Metaplasic	III	+++	0.05 0.02	Allethrin Deltamethrin
Complex	Metaplasic	III	0		--------------
Simple	Ductal	I	++		--------------
Simple	Ductal infiltrative	III	+		--------------
Complex	Metaplasic	III	+++	0.03	Cypermethrin
Complex	Metaplasic	III	0		--------------
Complex	Metaplasic	III	0		---------------

## Discussion

It is very little the number of studies connecting steroid hormone receptors and mammary tumors in female dogs. However, some human and canine carcinogenic hormone aspects seem to be similar. Toxicological and Epidemiological studies have shown that steroid hormones and synthetic derived trigger the development of mammary gland, suggesting that they may play an important role in the canine mammary tumor pathogenesis [20].

**Figure 5:**
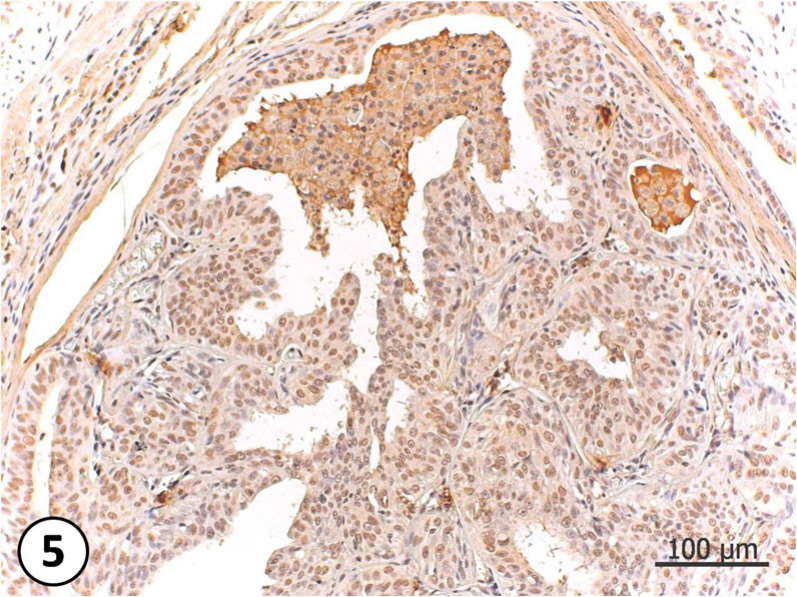
**Estrogen receptors' Immunohistochemical picture**. Observe the nuclear pattern marking. Simple carcinoma, grade III, +++ for ER. 200X.

**Figure 6:**
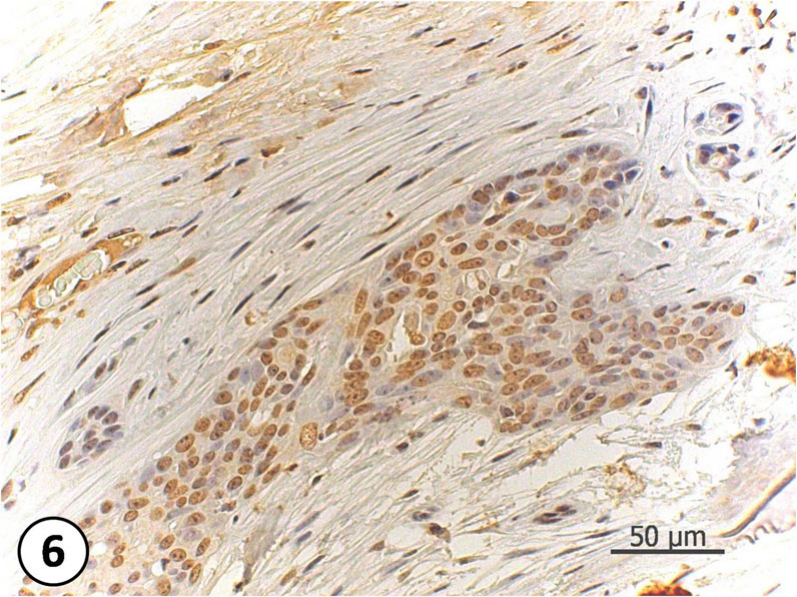
**Estrogen receptors' Immunohistochemical picture**. Complex carcinoma, grade III, +++ for ER. 400X.

**Figure 7:**
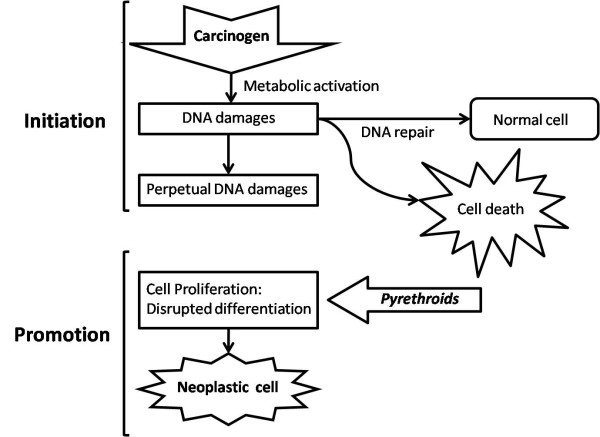
**Mammary carcinogenesis in the presence of pyrethroids**.

It is said that in the hormonal carcinogenesis, unlike the one induced by virus or chemical agents, the cell proliferation does not need a specific triggering agent. Hormones induce the cell proliferation together with genetic mutations that will give rise to neoplasic cells [21]. However, Carreño et al. (1999) [22], shows that the hormone role in the carcinogenesis is restricted to the proliferation of cells that have already been changed by other carcinogens. Specific genes involved in the development of hormone-dependent neoplasias are still unknown. Nevertheless, it is believed that oncogenes, genes that are tumor suppressors and the genes of DNA repairment are involved in the hormonal carcinogenesis, especially in the one induced by sexual steroids [23]. Having said that, this study has shown that the detected contaminants were present in more aggressive tumors (degree III and +++ for estrogen receptors- Fig.5 and 6). Even though the literature showing that being positive for estrogen receptors may result in good prognostic for women [24], the results from this preliminary study suggest that the presence of pyrehroids in the peritumoral fat may have triggered the local estrogenic effect and thus triggered higher proliferation of tumor cells. Scheme below (Fig. 7) shows the carcinogenesis of mammary tumor and the role of pyrethroids in the proliferation of tumors.

Epidemiological studies and tests on rodents have demonstrated the influence of environmental contaminants on neoplasia development, including breast cancer [10,25,26]. Garey et al, (1998) [27] emphasized that pyrethroids can induce a breakdown in the homeostasis of hormones such as estrogens and indirectly influence cell proliferation or apoptosis, either increasing or diminishing them in mammary epithelial cells, thereby triggering the neoplastic process. Results obtained in the present study detected the presence of 33.3% of pyrethroid pesticides in adipose tissue of female dogs with mammary carcinoma.

In order to improve results, and collection of data, our group is still researching such environmental contamination, increasing the number of samples as well as including samples from control animals.

## Competing interests

The authors declare that they have no competing interests.

## Authors' contributions

FHEA have made substantial contributions to the conception, design and collection of samples. FCF have made substantial contributions to the conception, design, interpretation of histopathological and immunohistochemical data, and critically revising it for important intellectual content. PROB have been involved in drafting the manuscript, tables, schemes and critically revising it for important intellectual content. DZB carried out the HPLC quantification and the interpretation of data. NSR conceived the study, and participated in its design and coordination, and also helped to draft the manuscript. All authors read and approved the final version of the manuscript.

## Supplementary Material

Additional file 1 Standard for HPLC of pyrethroids and adipose tissue with pyrethroid contamination. Graphics presenting the standard data for HPLC of pyrethroids and adipose tissue with pyrethroid contamination. Click here for file
