# Supplementary material for: Malignant mammary tumor in female dogs: environmental contaminants
Source: Diagn Pathol. 2010 Jun 30;5:45. doi: 10.1186/1746-1596-5-45 (PMC2909155; doi:10.1186/1746-1596-5-45)
Supplement: Additional file 1 — Standard for HPLC of pyrethroids and adipose tissue with pyrethroid contamination. Graphics presenting the standard data for HPLC of pyrethroids and adipose tissue with pyrethroid contamination. [file 1746-1596-5-45-S1.PDF]

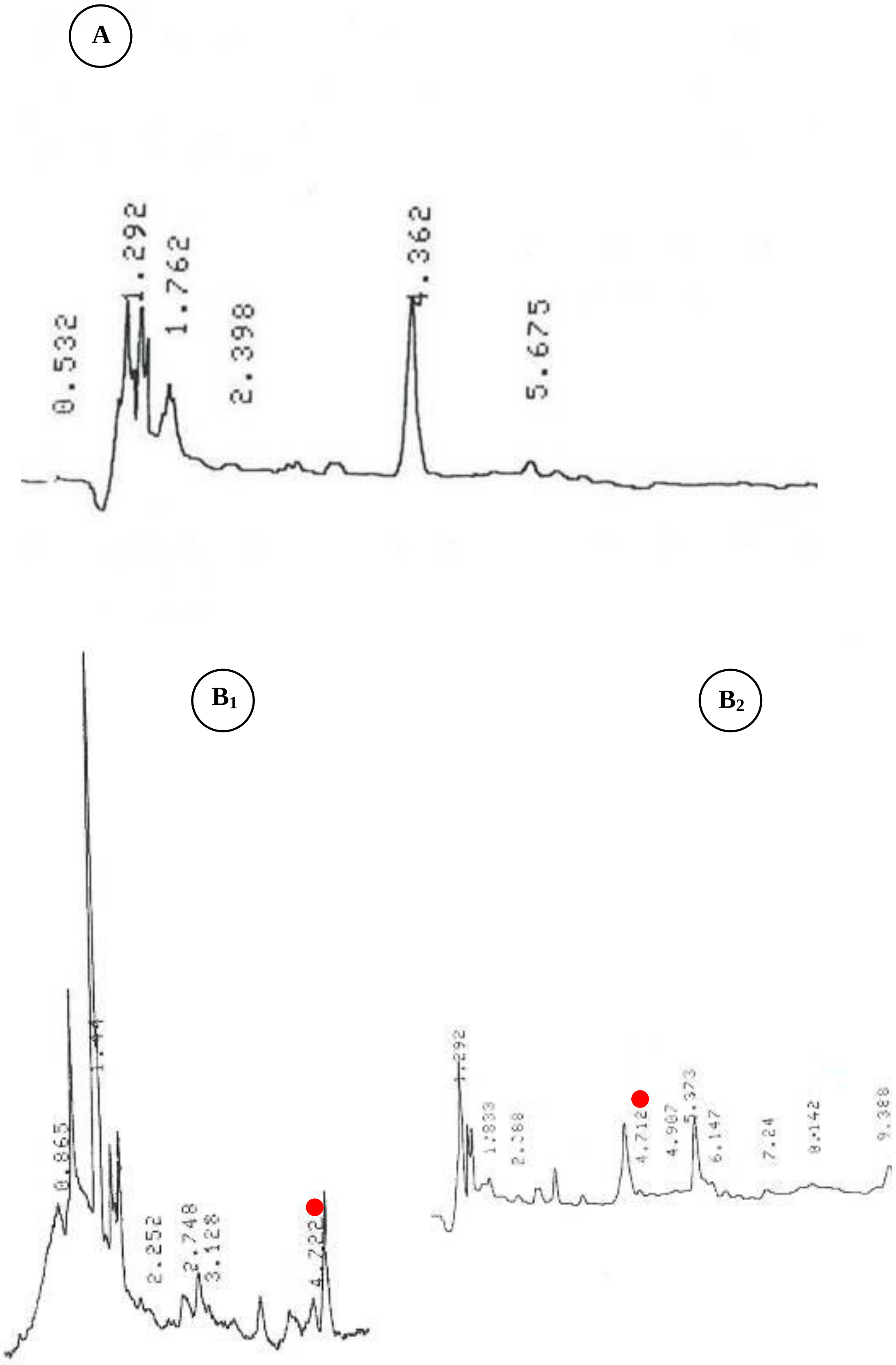
A
0.532
1.292
1.762
2.398
4.362
5.675
B1
0.865
2.252
2.748
3.128
4.722
B2
4.712
4.907
5.373
6.147
7.24
8.142
9.388

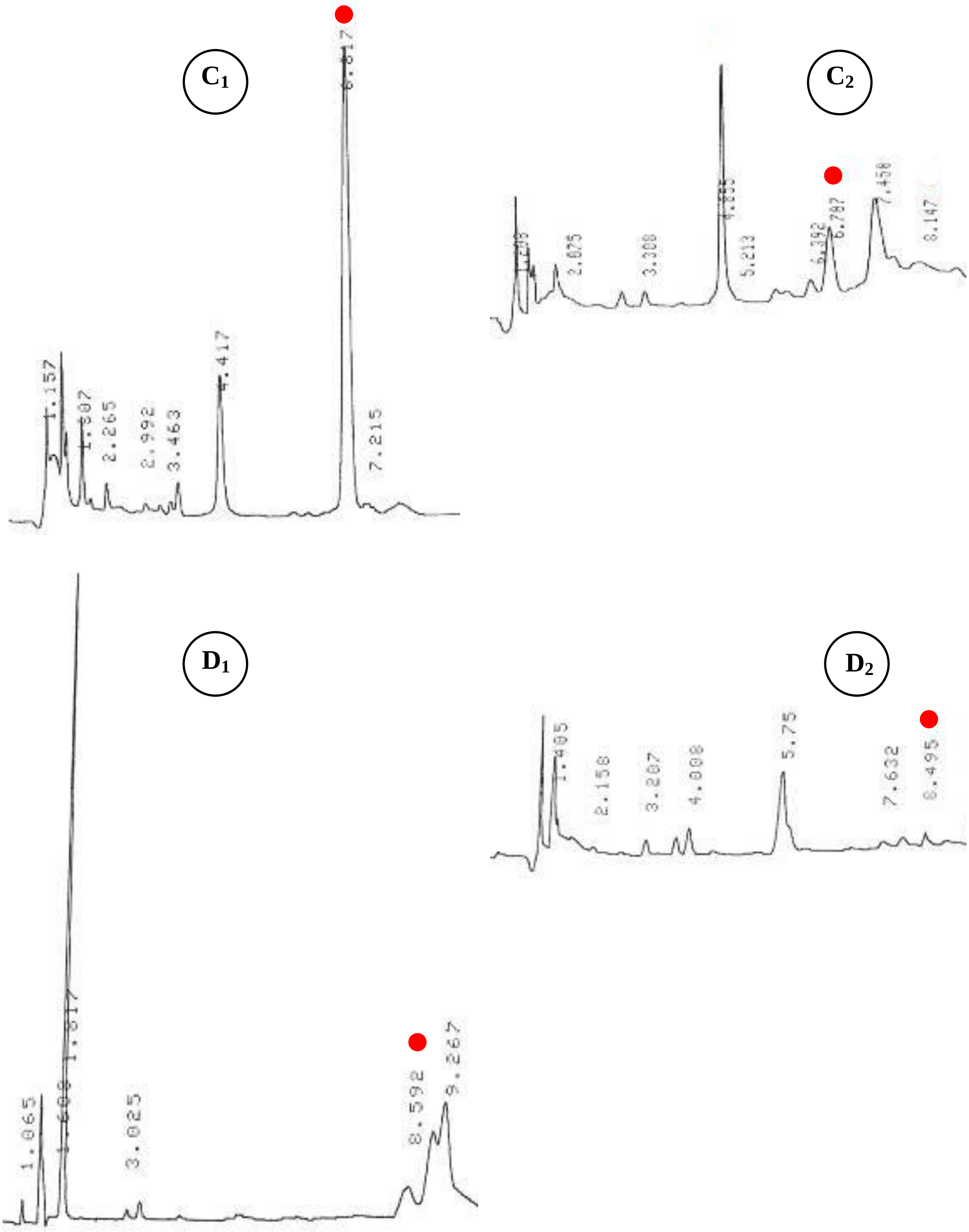
C1
1.157
2.265
2.992
3.463
4.417
7.215
C2
2.875
3.308
5.213
6.392
6.787
7.458
8.147
D1
1.065
1.817
3.025
8.592
9.267
D2
1.405
2.158
3.207
4.008
5.75
7.632
8.495

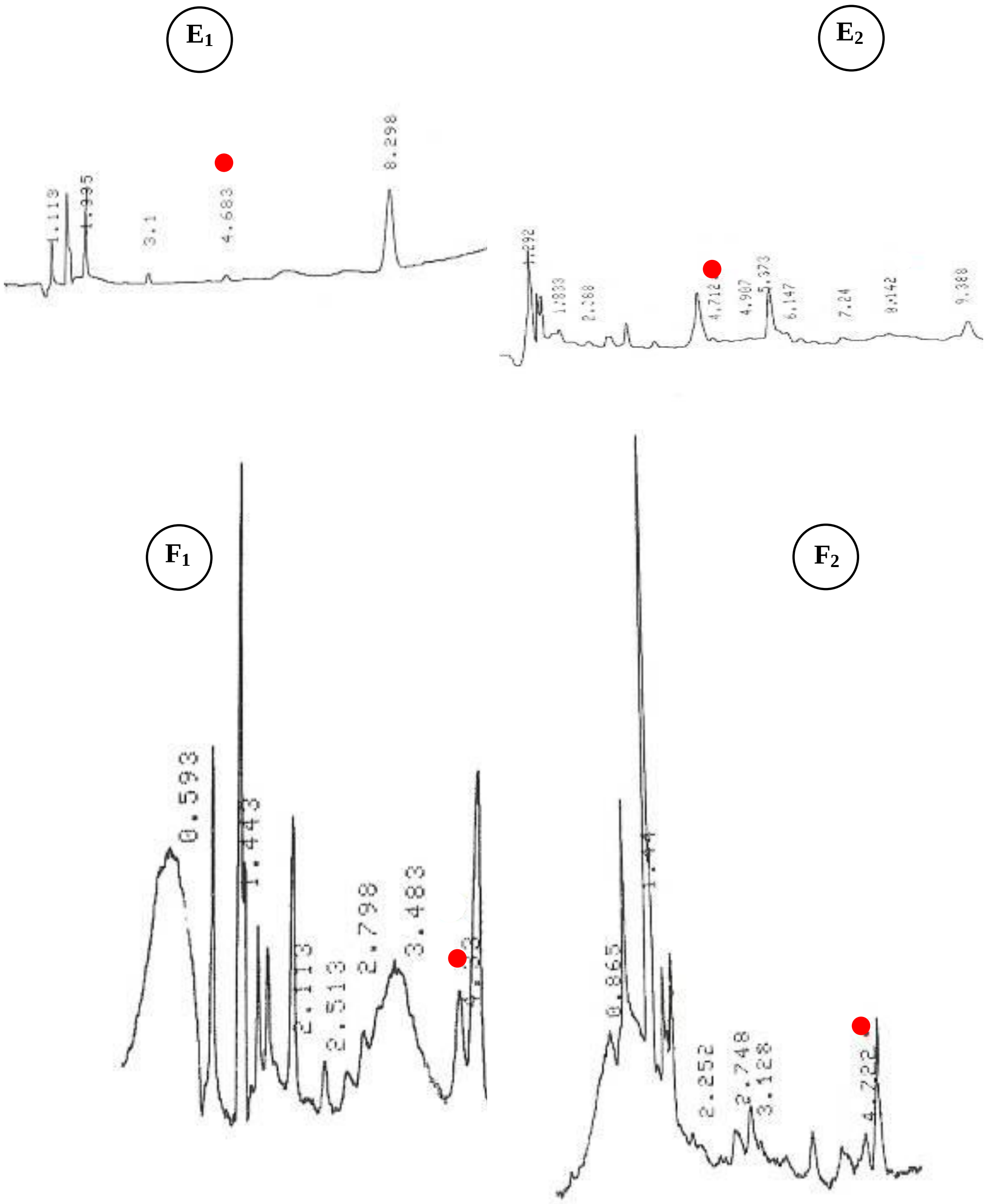

Graphic 1 – High Performance Liquid Chromatography – HPLC. A: Chromatogram of adipose tissue control adjacent to mammary carcinoma in female dog. B1, C1, D1, E1 and F1: Standard chromatogram of pyrethroid insecticide of Allethrin, Cyalothrin, Cypermethrin, Deltamethrin and Tetramethrin, respectively, to detect and identify contamination levels of adipose tissue adjacent to mammary tumor of female dog. The arrow indicates the detection peak. B2, C2, D2, E2 and F2: Chromatogram of identification of the pyrethroid insecticide - Allethrin, Cyalothrin, Cypermethrin, Deltamethrin and Tetramethrin, respectively, in samples of adipose tissue adjacent to mammary tumor in female dog. The red ball indicates the retention time.
